# Morphometric similarity networks discriminate patients with lumbar disc herniation from healthy controls and predict pain intensity

**DOI:** 10.3389/fnetp.2022.992662

**Published:** 2022-10-25

**Authors:** Lili Yang, Andrew D. Vigotsky, Binbin Wu, Bangli Shen, Zhihan Yan, A. Vania Apkarian, Lejian Huang

**Affiliations:** ^1^ Department of Radiology, The Second Affiliated Hospital and Yuying Children’s Hospital of Wenzhou Medical University, Wenzhou, Zhejiang, China; ^2^ Departments of Biomedical Engineering and Statistics, Northwestern University, Evanston, IL, United States; ^3^ Department of Pain Medicine, The Second Affiliated Hospital and Yuying Children’s Hospital of Wenzhou Medical University, Wenzhou, Zhejiang, China; ^4^ Department of Neuroscience, Feinberg School of Medicine, Northwestern University, Chicago, IL, United States; ^5^ Center for Translational Pain Research, Feinberg School of Medicine, Northwestern University, Chicago, IL, United States

**Keywords:** morphometric similarity networks, lumbar disc herniation, chronic pain, pain state discrimination, pain intensity prediction

## Abstract

We used a recently advanced technique, morphometric similarity (MS), in a large sample of lumbar disc herniation patients with chronic pain (LDH-CP) to examine morphometric features derived from multimodal MRI data. To do so, we evenly allocated 136 LDH-CPs to exploratory and validation groups with matched healthy controls (HC), randomly chosen from the pool of 157 HCs. We developed three MS-based models to discriminate LDH-CPs from HCs and to predict the pain intensity of LDH-CPs. In addition, we created analogous models using resting state functional connectivity (FC) to perform the above discrimination and prediction of pain, in addition to comparing the performance of FC- and MS-based models and investigating if an ensemble model, combining morphometric features and resting-state signals, could improve performance. We conclude that 1) MS-based models were able to discriminate LDH-CPs from HCs and the MS networks (MSN) model performed best; 2) MSN was able to predict the pain intensity of LDH-CPs; 3) FC networks constructed were able to discriminate LDH-CPs from HCs, but they could not predict pain intensity; and 4) the ensemble model neither improved discrimination nor pain prediction performance. Generally, MSN is sensitive enough to uncover brain morphology alterations associated with chronic pain and provides novel insights regarding the neuropathology of chronic pain.

## 1 Introduction

A new technique, morphometric similarity (MS) (Seidlitz et al., 2018), estimates the similarity of multiple morphometric features across brain regions using multimodal MRI data. MS can account for 40% of between-subject variability of IQ across 308 participants (Seidlitz et al., 2018), highlighting its ability to capture brain correlates of inter-individual differences. Moreover, MSs from three independent studies of schizophrenia patients were associated with both patients’ psychosis and genes (Morgan et al., 2019). Since this novel approach reflects anatomical “networks” from both histological and axonal connectivity similarity within an individual human brain, these results suggest morphometric similarity may be a promising approach to detect the morphometric differences in patients with chronic pain (CP).

Lumbar disc herniation (LDH) is a common back pathology that evokes back and/or leg pain, in which the annulus fibrosus allows the nucleus pulposus to move into the spinal canal (Kreiner et al., 2014; Amin et al., 2017). However, not all LDH patients report satisfactory pain relief after medical treatments (Sinmaz and Akansel 2021; Wakaizumi et al., 2021). Thus, LDH is a major contributor to CP, which limits physical activity, induces psychological distress, reduces social functional capacity, and, in turn, remarkably diminishes patients’ quality of life (Goldberg and McGee 2011; Kose and Hatipoglu 2012). The neural mechanisms that underlie the development of CP are poorly understood, thereby restricting clinicians from further improving medical treatments for relieving pain.

We have investigated functional reorganization of LDH patients with chronic pain (LDH-CP) by analyzing their functional connectivity (FC) networks. LDH-CPs’ whole-brain FC networks are disrupted compared with healthy controls (HCs), and these disruptions are correlated with pain intensity (Huang et al., 2019). However, we were unable to find reliable morphometric biomarkers for LDH-CPs, suggesting that 1) unlike other CP conditions, morphological properties are unaffected by LDH (Apkarian et al., 2004; Schmidt-Wilcke et al., 2005; Kuchinad et al., 2007; Schmidt-Wilcke et al., 2007; Geha et al., 2008; Kim et al., 2008; Schweinhardt et al., 2008; Valfre et al., 2008; Blankstein et al., 2010; Tu et al., 2010; Tu et al., 2013; Cauda et al., 2014; Hubbard et al., 2014; Kim et al., 2015; Lee et al., 2015; Coppieters et al., 2016; Sugimine et al., 2016; Preissler et al., 2017; Liao et al., 2018; Tatu et al., 2018; Bhatt et al., 2019; Barroso et al., 2020), or 2) our measures were not sensitive enough (Good et al., 2001; Baliki et al., 2011). Thus, it is imperative to find a more sensitive technique to reconcile the aforementioned possibilities, and the novel method of morphometric similarity is a good candidate for exploration.

In this paper, we evenly allocated 136 LDH-CPs to exploratory and validation groups with matched HCs. We first constructed a MS matrix for individual subjects. Second, we developed three MS-based models to discriminate LDH-CPs from HCs and compared their performance. Third, we constructed a PCA-LASSO-GLM model to predict pain intensity of LDH-CPs. Fourth, we created a model based on FC to perform the above discrimination and the prediction and the performance was compared. Finally, we investigated if an ensemble model, combining MS and FC, could improve performance.

## 2 Methods

### 2.1 Participants

One hundred thirty six LDH-CPs were recruited [87 males, 49 females; age (mean ± SD) = 44 ± 12 years old], diagnosed by medical history, physical examination, and consistent MRI assessment confirmed independently by two radiologists (Kreiner et al., 2014), with chronic pain that persisted for at least 12 weeks; 157 healthy volunteers were recruited as healthy controls (HC) (77 males, 80 females; age (mean ± SD) = 40 ± 14 years old) without chronic pain for at least the last 52 weeks (1 year). Participants were excluded if they 1) were less than 18 or greater than 85 years old; 2) reported a history of head injury and/or cerebral disease (e.g., stroke or encephalopathy); 3) had diabetes or a psychiatric disease; 4) reported a history of brain neurosurgical procedures and/or epilepsy; 5) were unable to cooperate (e.g., psychogenic or cognitively impaired); 6) reported pregnancy, drug dependence, or drug abuse; 7) were not suitable for MRI scan. All participants were scanned for T1-weighted structural image (T1), resting-state functional MRI (RS-fMRI) and diffusion tensor imaging MRI (DTI).

This study was approved by the Institutional Review Board of the Second Affiliated Hospital and Yuying Children’s Hospital of Wenzhou Medical University, China, and all participants signed a written informed consent.

### 2.2 Study design

The 136 LDH-CPs were evenly allocated to either an exploratory group (Discovery) or a validation group (Validation) with matched demographics and pain-related data (Table 1). For each group, 68 HCs were randomly chosen from the pool of 157 HCs with matched demographics to its corresponding LDH-CPs (Table 1). Data exploration was performed in Discovery (68 HCs vs. 68 LDH-CPs) and the results acquired in Discovery were tested for corroboration in Validation group (68 HCs vs. LDH-CPs). Education was categorized to low education (middle school or below) and high education (high school or above). The participants were inquired their smoking status (Yes/No), daily alcohol and exercise frequency (0: Not at all; 1: Sometimes; 2: Always). For each demographic characteristic (age, sex, BMI, education level, smoking and alcohol status, and exercise), a two-way ANOVA was performed to assess whether either of the two independent variables (group: Discovery vs. Validation; pain status: HC vs. LDH-CP) or their interaction are statistically significant. For each pain-related characteristic (pain intensity and duration), a one-way ANOVA was performed to assess the significance of the difference between the Discovery and Validation groups. For those statistically significant variables, a Turkey post hoc test was performed for pairwise multiple comparisons.

**TABLE 1:** Demographic and pain-related characteristics of HCs and LDH-CPs in the Discovery and the Validation groups. Two-way and one-way ANOVAs were performed for each demographic and pain-related variables, respectively. The *p* values in the table corresponds to that of group (Discovery vs. Validation), pain status (HC vs. LDH-CP), and their interaction. y/o: years old; SD: standard deviation; BMI: body mass index; wks: weeks. #: the *p* value of pain duration was calculated from log_10_ (pain duration) by a Wilcoxon signed-sum test.

Variables	Discovery		Validation		*p* values		
	HC (*n* = 68)	LDH-CP (*N* = 68)	HC (*n* = 68)	LDH-CP (*N* = 68)			
Age (y/o) mean (SD)	43.65 (13.77)	43.57 (14.38)	44.01 (12.34)	44.04 (11.32)	0.989	0.791	0.974
Male n (%)	41 (60.29)	43 (63.23)	43 (63.23)	44 (63.76)	0.901	0.535	0.901
BMI mean (SD)	22.23 (2.62)	23.96 (3.04)	23.39 (3.15)	23.60 (3.54)	0.290	0.010	0.045
Low education n (%)	24 (35.29)	44 (64.71)	28 (41.18)	43 (63.23)	0.709	0.000	0.533
Current smoking n (%)	9 (13.23)	20 (29.41)	11 (16.18)	19 (27.94)	0.882	0.005	0.657
Alcohol mean (SD)	0.87 (0.91)	0.84 (0.86)	0.93 (0.90)	0.85 (0.89)	0.734	0.634	0.838
Exercise mean (SD)	1.00 (0.72)	0.85 (0.89)	1.13 (0.73)	0.88 (0.87)	0.408	0.043	0.598
Pain intensity mean (SD)	—	49.33 (20.44)	—	50.35 (21.02)	—	0.766	—
Pain duration (wks) median, min, max	—	80, 12, 664	—	104, 12, 1040	—	0.976^#^	—

### 2.3 MRI scanning parameters

Subjects were scanned on a 3 T GE-Discovery 750. T1 images were acquired with following parameters: voxel size = 1 × 1 × 1 mm^3^; TR/TE = 7.7/3.4 ms; flip angle = 12°; in-plane resolution = 256 × 256; field of view = 256 × 256 mm^2^; slices per volume = 176. RS-fMRI images were acquired with the following parameters: voxel size = 3.4375 × 3.4375 × 3.5 mm^3^; TR/TE = 2500/30 ms; flip angle = 90°; in-plane resolution = 64 × 64; field of view = 220 × 220 mm^2^; number of volumes = 230; slices per volume = 42, which covers the whole brain from the cerebellum to the vertex. DTI images were acquired with the following parameters: voxel size = 2 × 2 × 2 mm^3^; TE/TR = 75/8000 ms; flip angle = 90^0^; in-plane resolution = 128 × 128; field of view = 256 × 256 mm^2^; slices per volume = 67; 2 shells with 23 directions (b-value = 1000 s/mm^2^) and 49 directions (b value = 2000 s/mm^2^); at the beginning of each shell, two images with b-value = 0 were scanned.

To minimize head motion, a head support system consisting of two foam pads positioned on either side of the head was used, along with earplugs for reducing scanner noise. Participants were instructed to keep their eyes open and to remain as still as possible during acquisition.

### 2.4 T1, RS-fMRI and DTI data preprocessing

After individual T1 image was visually inspected for motion artifacts, FreeSurfer v6.0 software (Fischl, 2012) was applied to perform T1 preprocessing and cortical reconstruction processes. Briefly, first, motion correction, conform, intensity normalization, and skull strip; second, volumetric registration and labeling; third, gray and white matter segmentation, smoothing, and inflation; and fourth, spherical mapping and registration, and cortical parcellation.

A similar preprocessing pipeline in (Huang et al., 2019) was applied to all RS-fMRI data. Briefly, it consisted of removal of the first 4 volumes (10 s) for magnetic field stabilization; motion correction; slice-time correction; intensity normalization; high-pass temporal filtering (0.008 Hz) for correcting low-frequency signal drift; nuisance regression of 6 motion vectors, signal-averaged overall voxels of the eroded white matter and ventricle region, and global signal of the whole brain; motion-volume censoring by detecting volumes with frame-wise displacement larger than 0.5 mm, derivative variance root mean square after Z-transformation larger than 2.3, and standard deviation after Z-transformation larger than 2.3, and scrubbing above detected (number of volume = *i*) and adjacent four volumes (*i* - 2, *i* - 1, *i* + 1, *i* + 2) (Power et al., 2012; Power et al., 2014); band-pass filtering (0.008–0.1 Hz) by applying a 4th-order Butterworth filter. All pre-processed RS-fMRI data were registered to the MNI152 template using a two-step procedure, in which the mean of preprocessed fMRI data was registered with a 7-degree-of-freedom affine transformation to its corresponding T1 brain (FLIRT); transformation parameters were computed by nonlinearly registering individual T1 brains to the MNI152 template (FNIRT). Combining the two transformations by multiplying the matrices yielded transformation parameters that normalized the pre-processed fMRI data to the standard space.

The two-shell DTI images were concatenated with corresponding combined b-values and b-vectors. Afterwards, DTI data were manually checked, volume by volume, for obvious artifacts. By utilizing FMRIB’s Diffusion Toolbox (FDT), eddy current and head motion correction was performed. A diffusion tensor model was fit at each voxel to generate voxel-wise fractional anisotropy (FA) and mean diffusivity (MD) (Alexander et al., 2007).

### 2.5 Construction of morphometric similarity matrix

**FIGURE 1:**
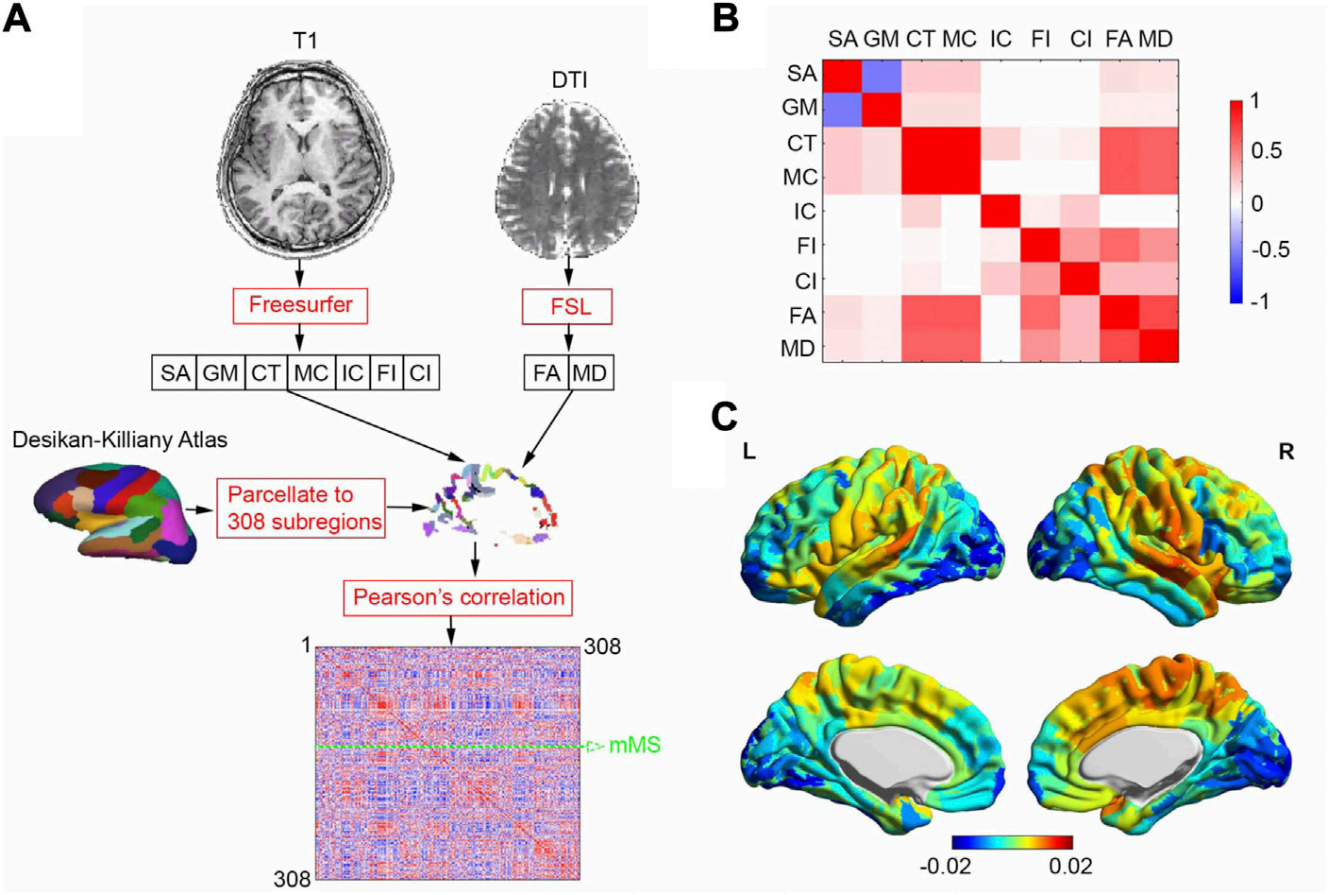
MSN generating process and averaged correlation coefficients between 9 subregional features and MSN. **(A)** MSN generating process for one subject. We analyzed T1 and DTI brain images using *Freesurfer* and *FSL* to generate 7 gray matter-related and 2 tractographic feature maps, respectively. The feature maps were projected onto a 308-subregion surface-cortical template derived from the Desikan-Killiay Atlas with approximately equal surface area, extracting 9 subregional features averaged on each subregion. A 308 × 308 morphometric similarity matrix was generated, consisting of pairwise Pearson correlation coefficients between the subregions. Mean morphometric similarity (mMS) of one of 308 subregions was calculated by averaging MSs over other 307 subregions (green dashed arrow line). **(B)** Averaged pairwise Pearson correlation coefficients between 9 subregional features over 68 HCs in Discovery group **(C)** Averaged regional morphometric similarity over 68 HCs in Discovery group, SA: surface area; GM: gray-matter volume; CT: cortical thickness; MC: mean curvature; IC: intrinsic curvature; FI: folding index; CI: curved index; FA: fractional anisotropy; MD: mean diffusivity.

As shown in Figure 1A, *Freesurfer* and *FSL* were used to analyze the T1 and DTI images to generate 7 gray matter-related (surface area, gray matter volume, cortical thickness, mean curvature, intrinsic curvature, folding index, and curved index) and 2 tractographic (fractional anisotropy and mean diffusivity) feature maps, respectively. We reduced the dimensionality of the analysis in a way that will leverage small-world properties in anatomical cortical networks (Romero-Garcia et al., 2012), which involved applying the Desikan–Killiany atlas (Desikan et al., 2006) to a 308-subregion template with approximately equal surface area (Seidlitz et al., 2018). Following this, the 9 feature maps were projected onto the 308-subregion template, extracting 9 subregional features averaged on each subregion. In the end, after each feature was zscored Pearson’s correlations were calculated between each pair of subregions to generate a 308 × 308 MS matrix. Mean MS (mMS) of each individual subregion was calculated by averaging the off-diagonal elements of a row or column in the similarity matrix (green dashed arrow line in Figure 1A). Averaged pairwise Pearson correlation coefficients between 9 subregional features over 68 HCs in the Discovery group was shown in Figure 1B. Note that the 2 tractographic features were derived from gray matter since the Desikan–Killiany atlas is a surface-cortical template.

### 2.6 Three models to discriminate pain state of LDH-CPs from HCs

Three models derived from morphometric similarity matrices were developed to discriminate LDH-CPs from HCs. The mMS-based univariable model was based on mMS, calculated by averaging MSs over other 307 subregions (green dashed arrow line in Figure 1A). This model assumed that the mMS of each of the 308 subregion is independent; thus, for each subregion, a *t*-statistic was calculated (age, sex, BMI, education level, alcohol, and exercise as covariates) to discern participants’ pain state (Discovery data; 2 states: pain for 68 LDH-CPs and no pain for 68 HCs; a matrix of 136 × 308). Only subregions that were statistically significant (*p* value < 0.05) in Discovery and replicated in Validation were regarded as a biomarker to discriminate LDH-CP patients from HCs.

The mMS-based multivariable model arose from the *β* estimated for each subregion. Logistic regression was independently used to estimate *β*_*j*_ for each subregion (*j* = 1, 2, …, 308), where the response variable ***y*** ([*y*_*1*_, *y*_*2*_, …, *y*_*136*_]^T^) was pain state (*y*_*i*_ = 1 for LDH-CPs and *y*_*i*_ = 0 for HCs, *i* = 1, 2, …, 136) and the predictor ***x*** ([*x*_*1*_, *x*_*2*_, …, *x*_*136*_]^T^) was mMS for subregion *j* in the Discovery group, with age, sex, BMI, education level, alcohol, and exercise set as covariates. For a given subject, the dot product of the ***β*** vector ([*β*_*1*_, *β*_*2*_, …, *β*_*308*_]^T^) and ***mMS*** vector ([*mMS*_*1*_, *mMS*_*2*_, …, *mMS*_*308*_]^T^) was the so-called “linear predictor” used to discriminate between groups.

The MSN-based multivariable model took advantage of the morphometric similarity network, assigning *β* to each edge (connectivity) in the upper/lower triangular matrix since the network is bidirectional. To overcome overfitting *β*, principal component analysis (PCA) was performed before fitting the logistic regression. Briefly, first, for each subject *i* (*i* = 1, 2, …, 136), the upper triangular matrix of each ***MSN***_*i*_ was vectorized to a row vector ***r***_*i*_ = ([*r*_*1*_, *r*_*2*_, …, *r*_*47278*_]); all ***r***_*i*_ were row-wise concatenated, generating a 136 × 47,278 matrix ***R***; second, PCA was performed on ***R*** to reduce dimensions, generating a 136 × 136 PCA matrix, ***R***_***PCA***_; third, logistic regression was used to estimate *β*_*PCAj*_ corresponding to each component of ***R***_***PCA***_(*:*, *j*) (*j* = 1, 2, …, 136) where the response ***y*** ([*y*_*1*_, *y*_*2*_, …, *y*_*136*_]^T^) was pain state (*y*_*i*_ = 1 for LDH-CPs and *y*_*i*_ = 0 for HCs, *i* = 1, 2, …, 136) and the columns of ***R***_***PCA***_ were regressors, with age, sex, BMI, education level, alcohol, and exercise set as covariates; fourth, we projected the principal component coefficients, ***β***_***PCA***_ ([*β*_*PCA1*_, *β*_*PCA*2_, …, *β*_*PCA136*_]^T^) back to ***β*** ([*β*_*1*_, *β*_2_, …, *β*_*47278*_]^T^) and transformed the result into an upper triangular matrix, ***β***_***upper***_. Similarly, the dot product of ***β***_***upper***_ and upper triangular of each subject’s ***MSN***_*j*_, defined a ${\hat{ps}}_{j}$ , served as the “linear predictor” of each subject’s pain state (LDH-CP vs. HC), thus generating a column vector of estimate of pain state, $\hat{ps}$ ([ ${\hat{ps}}_{1\;}{,\;\hat{ps}}_{2\;},\;{...,\hat{ps}}_{136}$ ]^T^). Considering that this decoding model is “backward” from which the visualization of activation brain is uninterpretable and even misleading, ***β*** was transformed to activation pattern **A** using the following equation, **A** = **Σ**_**R**_ * ***β***/var ( $\hat{ps}$ **)**, where **Σ**_**R**_ is the covariance matrix of ***R***, and var ( $\hat{ps}$ **)** is the variance of $\hat{ps}$ (Haufe et al., 2014; Zhou et al., 2021).

To assess the performance of the three models for discriminating LDH-CPs from HCs, the models developed in the Discovery group were corroborated in the Validation group and the area under the receiver operating characteristic curve (AUC) was calculated and compared. In addition, to examine the sampling variability, the Discovery and Validation groups were switched, and the discriminating performance was reassessed.

### 2.7 PCA-LASSO-GLM for predicting pain intensity of LDH-CPs

The least absolute shrinkage and selection operator (LASSO) (Tibshirani, 1996) was used with principal components regression to predict pain intensity of LDH-CPs. This involved applying PCA to the predictor matrix before fitting a regression with L_1_ regularization. Briefly, first, for each subject of LDH-CP *i* (*i* = 1, 2, …, 68), the upper triangular matrix of each ***MSN***_*i*_ was vectorized to a row vector ***r***_*i*_ = ([*r*_*1*_, *r*_*2*_, …, *r*_*47278*_]); all ***r***_*i*_ were row-wise concatenated, generating a 68 × 47,278 matrix ***R***; second, PCA was performed on ***R*** to reduce the number of dimensions, generating a 68 × 68 PCA matrix, ***R***_***PCA***_; third, when ***y*** ([*y*_*1*_, *y*_*2*_, …, *y*_*68*_]^T^) was pain intensity and the columns of ***R***_***PCA***_ were regressors, with age, sex, BMI, education level, alcohol, and exercise set as covariates, we used MATLAB’s *lassoglm* function with leave-one-out cross-validation on the training data of LDH-CPs in the Discovery group, choosing the hyperparameter *λ* with minimum expected deviance; fourth, with the specified hyperparameter *λ*, *lassoglm* was run again to estimate *β*_*PCAj*_ corresponding to each component of ***R***_***PCA***_(*:*, *j*) (*j* = 1, 2, …, 68); finally, we projected the principal component coefficients, ***β***_***PCA***_ ([*β*_*PCA1,*_ *β*_*PCA*2, … ,_ *β*_*PCA68*_]^T^) back to ***β*** ([*β*_*1,*_ *β*_2_, …, *β*_*47278*_]^T^) and transformed the estimates to an upper triangular matrix, ***β***_***upper***_. Thus, for a given subject *j*, the dot product of ***β***_***upper***_ and upper triangular of its ***MSN***_*j*_, defined as ${\hat{NRS}}_{j}$ , would predict their pain intensity, $\hat{NRS}$ ([ ${\hat{NRS}}_{1\;}{,\;\hat{NRS}}_{2\;},\;{...,\hat{NRS}}_{68}$ ]^T^). To assess the model’s performance, we applied the model to the Validation group and calculated the Pearson correlation coefficients between the dot product and observed pain. Similar to **2.6**, ***β*** was also transformed to activation pattern **A** as well for interpretation using the following equation, **A** = **Σ**_**R**_ * ***β***/var ( $\hat{NRS}$ **)**, where **Σ**_**R**_ is the covariance matrix of ***R***, and var ( $\hat{NRS}$ **)** is the variance of $\hat{NRS}$ .

### 2.8 Functional connectivity vs. morphometric similarity

On the same 308-subregin template, brain FC was estimated from RS-fMRI data, from which two models were developed to discriminate LDH-CP patients from HCs and to predict pain intensity of LDH-CP patients. Briefly, first, BOLD signals were extracted by averaging the BOLD signals from each subregion; second, a correlation matrix (308 × 308) was generated by calculating subregion-wise Pearson correlation coefficients of the BOLD signals; third, mean FC (mFC) of individual subregion was calculated by averaging the off-diagonal elements of a row or column in the correlation matrix; fourth, the same procedures performed in mMS and MSN (Section 2.6 and Section 2.7) were applied to mFC and FC network (FCN) and model performances were compared.

### 2.9 Ensemble model with morphometric features and resting-state signals

We evaluated performance of an ensemble model that combines both morphometric features and BOLD signals for discriminating group membership and predicting pain intensity of LDH-CPs. Briefly, for each subregion, the 9 morphometric features and averaged BOLD signals from the subregion were independently *z*-scored and concatenated to one vector; second, the weight of morphometric similarity, *w*_*ms*_, varied from 0 to 1 (0: no MS effect; 1: no FC effect). The weight vector of the mixture, ***w***, defined as $w={\left[\frac{\left[{\;w}_{ms\;}w_{ms\;}\ldots \;w_{ms\;}\right]}{9}\;\frac{\left[{\;1-w}_{ms\;}1-w_{ms\;}\ldots \;{1-w}_{ms\;}\right]}{Num.\;of\;Volumes}\right]}^{T}$ , was used to generate a weighted correlation matrix (MATLAB function *weightedcorrs*); third, for each *w*_*ms*_, we applied the same procedures of MSN model (see Section 2.6 and Section 2.7) to this ensemble model; finally, we verified the model using the Validation group, as described for the other models.

## 3 Results

### 3.1 Demographics and pain characteristics of LDH-CPs in Discovery and Validation

Table 1 summarizes the pain characteristics of the LDH-CPs. Mean pain intensity (0–100; 0 = no pain, 100 = worst pain imaginable) was 49/100 and 50/100 with a standard deviation (SD) of 20 and 21 for patients in Discovery and Validation groups, respectively (*p* = 0.766). Median pain duration was 80 and 104 weeks, and the range of pain duration was between 12 and 644 weeks and 104 weeks and between 12 and 1040 weeks for patients in Discovery and Validation groups, respectively (*p* = 0.976, calculated using log_10_ (pain-duration)). For LDH-CPs, the groups also had similar means for all demographic variables (age, sex, BMI, education level, smoking and alcohol status, and exercise). In addition, there was a statistically significant difference between HCs and LDH-CPs for BMI (*p* = 0.01), education level (*p* < 0.001), smoking status (*p* = 0.005), and exercise (*p* = 0.043) and significant interaction of BMI between HCs and LDH-CPs (*p* = 0.045).

### 3.2 Mean morphometric similarity

Averaged regional morphometric similarity of 68 HCs (Figure 1C) revealed that subregions were relatively similar to temporal association cortex and relatively distinct from occipital cortex, replicating the findings of (Seidlitz et al., 2018).

Compared with HCs, morphometric similarity of LDH-CPs in the Discovery group was increased in left rostral middle frontal cortex (uncorrected *p* = 0.025; Supplementary Table S1 red row) and left middle temporal cortex (uncorrected *p* = 0.011; Supplementary Table S1 red row) and decreased in right fusiform cortex (uncorrected *p* = 0.049; Supplementary Table S1 blue row) and right supramarginal cortex (uncorrected *p* = 0.017; Supplementary Table S1 blue row). Thus, morphometric similarity was able to uncover cortical structural differences in LDH-CPs. Note that there did not exist significantly different subregions after Bonferroni multiple-comparison correction.

### 3.3 Discrimination of LDH-CPs from HCs

Three models derived from morphometric similarity were developed to discriminate LDH-CPs from HCs. The mMS-based univariable model revealed that left rostral-middle-frontal-part8 [(−24, 56, 14); Supplementary Figures S1A,B] was the only subregion mMS that statistically significantly discriminated the pain state of LDH-PCs in the Discovery group (*t*_134_ = 2.25, *p* = 0.025, Supplementary Figure S1C left) and the result was replicated in the Validation group (*t*_134_ = 2.01, *p* = 0.047, Supplementary Figure S1C right).

**FIGURE 2:**
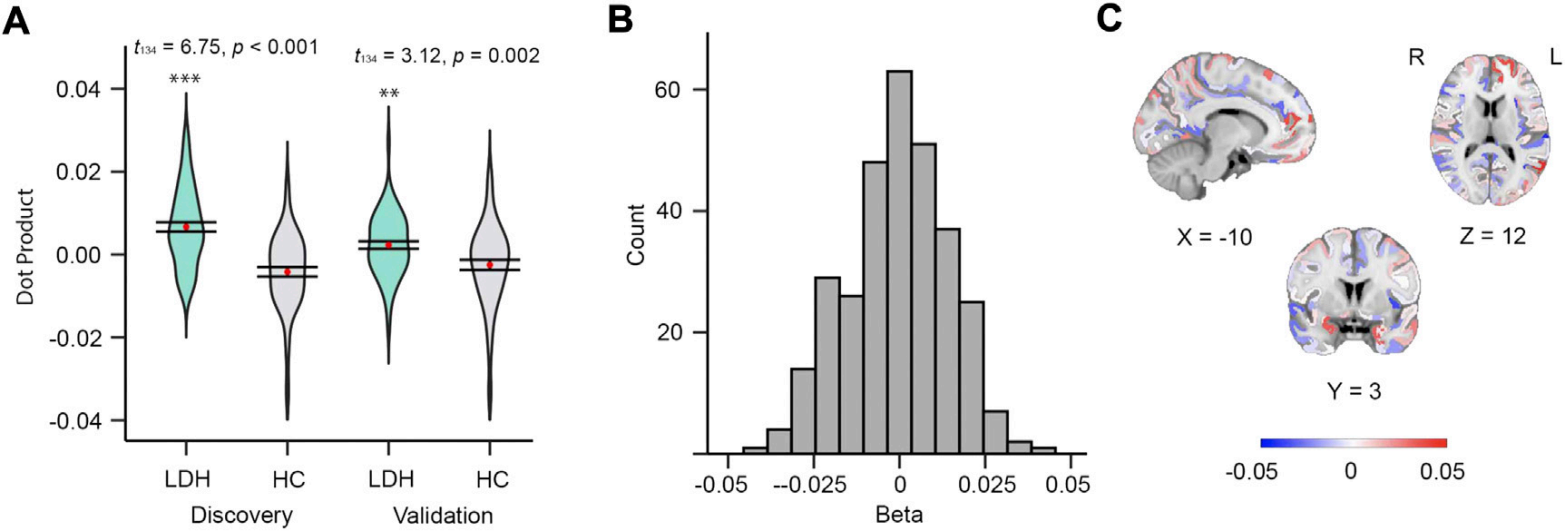
Mean MS of 308 subregions can discriminate patients from controls. **(A)** Violin plots of dot products in discovery (left, *t*_134_ = 6.75, *p* < 0.001) and validation (right, *t*_134_ = 3.12, *p* = 0.002) groups. **(B)** Histogram of betas from the 308 subregions derived from discovery group. **(C)** Beta map in MNI152 space.

The mMS-based multivariable model, which estimated the *β* of each of 308 subregions, also discriminated between groups (Discovery: *t*_134_ = 6.75, *p* < 0.001; Validation: *t*_134_ = 3.12, *p* = 0.002, Figure 2A); the distribution of *β*s and their cortical locations are shown in Figures 2B,C, respectively; Supplementary Table S1 illustrates the details of the top 10% *β*s (hemisphere, subregion location, *β* value, and its centroid coordinates). The top 10% of *β*s (absolute value) were from the frontal, fusiform, temporal, lingual, insula, cingulate, parietal, and precuneus cortex.

**FIGURE 3:**
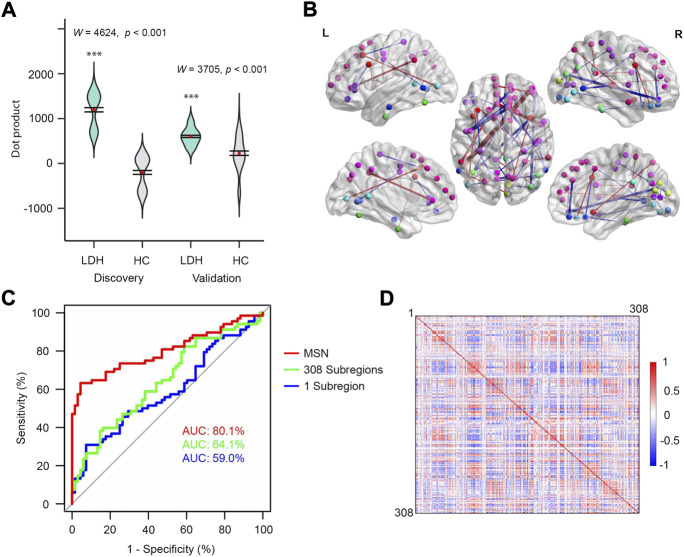
MSN performed best for discriminating patients from controls. **(A)** Violin plots of dot products in discovery (left, *W* = 4,624, *p* < 0.001) and validation (right, *W* = 3,705, *p* < 0.001) groups. Wilcoxon-Mann-Whitney test was performed for both groups. **(B)** The top 0.1% MSN model. Nodes indicate centroids of the 308 subregions and the nodes with identical color were from the same region of Desikan-Killiay Atlas. The size of each edge reflects the beta value for the two subregions and the red and blue colors represent positive (Pain: 1 and No Pain: 0) and negative beta values, respectively. **(C)** ROC curves of three models for validation data. **(D)** MS correlation coefficients averaged over 68 HCs (upper diagonal matrix) and 68 LDH-CPs (lower diagonal matrix) in discovery group.

The MSN-based multivariable model was able to distinguish LDH-CPs from HCs (Discovery: *W* = 4,624, *p* < 0.001; Validation: *W* = 3,705, *p* < 0.001, Figure 3A). Wilcoxon-Mann-Whitney test was performed for both groups because the distribution of results (Discovery group) was not normal. The top 0.1% of linkages are illustrated in Figure 3B and detailed in Supplementary Table S2, respectively, which covered linkages between occipital, cuneus, superior and middle frontal, precuneus, anterior cingulate, insula, pre- and postcentral, and middle temporal cortex. Thus, all three MS-based models were able to discriminate LDH-CPs from HC and they provide additional information to increase our understanding of the neuropathology in LDH-CP patients.

In addition, as shown in Figure 3C, after the models were developed using the Discovery dataset, they tested in the Validation dataset by calculating the AUC of three models. The MSN-based model had the best performance as compared to the other two (AUC = 80.1% vs. 59.0% and 64.1%). In this case, the more information included in the model, the better prediction accuracy for the model, indicating the model was not overfit. Moreover, sampling variability did not affect the performance, which was observed in Supplementary Figure S2 (AUC 79.4%, 60.1%, and 59.8% for above 3 models) after the Discovery and Validation groups were switched.

### 3.4 Prediction of pain intensity of LDH-CPs

**FIGURE 4:**
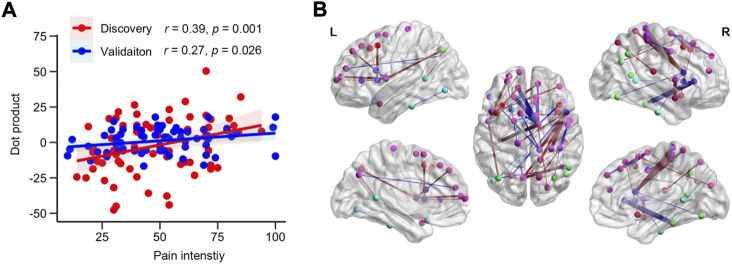
MSN can predict pain intensity of LDH-PC patients. **(A)** Scatter plot of the relationship between dot products and pain intensity of patients in the Discovery (*r* = 0.39, *p* = 0.001, *R* ^2^ = 0.15, RSME = 19.0) and Validation groups (*r* = 0.27, *p* = 0.026, *R* ^2^ = 0.07, RSME = 20.4). **(B)** Visualization of the top 0.1% of beta map of MSN. Nodes indicated centroid brain locations of 308 subregions and the nodes with identical color are from the same region from the Desikan-Killiay Atlas. The size of each edge reflects the beta value for the two subregions and the red and blue colors represents positive and negative beta value, respectively.

Figure 4A depicts the relationship between the dot product (linear predictor) and the pain intensity from LDH-CPs in the Discovery (*r* = 0.39, *p* = 0.001, *R* ^2^ = 0.15, RSME = 19.0) and the Validation (*r* = 0.27, *p* = 0.026, *R* ^2^ = 0.07, RSME = 20.4) groups. The top 0.1% of linkages between two subregions and their information is illustrated in Figure 4B and detailed in Supplementary Table S3, respectively, which covered linkages between superior, middle, and lateral-orbital frontal, precuneus, isthmus cingulate, insula, inferior parietal, pars opercularis, precentral, and inferior temporal cortex. Thus, with a PCA-LASSO-GLM algorithm, MSN could predict pain intensity of LDH-CPS.

### 3.5 FC discriminating pain state of LDH-PC patients from HCs

**FIGURE 5:**
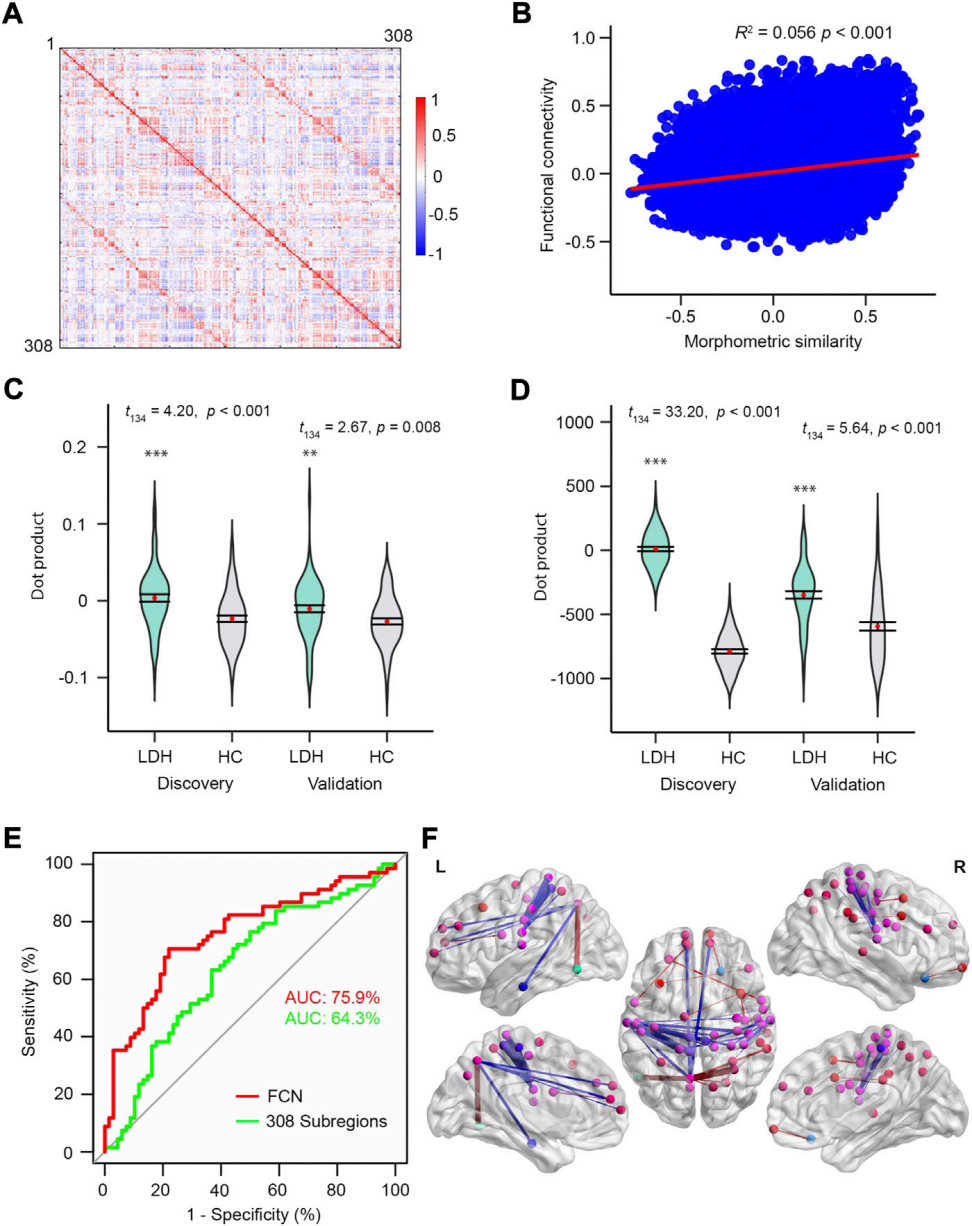
FC on the same subregions can discriminating patients from controls. **(A)** FC correlation coefficients averaged over 68 HCs in the discovery group. **(B)** The relationship between MS and FC averaged over HCs in discovery group. MS and FC only shared 5.6% of covariance. Visualization of the top 0.1% FCN. **(C)** and **(D)** Violin plots of dot products in Discovery (left, *t*_134_ = 4.20, *p* < 0.001; *t*_134_ = 33.20.20, *p* < 0.001) and Validation (right, *t*_134_ = 2.67, *p* = 0.008; *t*_134_ = 5.64, *p* < 0.001) groups for mean FS and FSN models, respectively. **(E)** ROC curves of the two models using the validation dataset. **(F)** Nodes of the 308 subregions and the nodes with identical color were from the same region of the Desikan-Killiay Atlas. The size of each edge reflects the beta value for the two subregions and the red and blue colors represents positive (Pain: 1 and No Pain: 0) and negative beta values, respectively.

Compared to morphometric similarity, FC was more bilaterally symmetric (Figures 3D, 5A). Although functional connectivity and morphometric similarity have unique patterns—sharing only 5.6% of their variance (Figure 5B) on the same 308-subregional template—both mFC- (Discovery: *t*_134_ = 4.20, *p* < 0.001; Validation: *t*_134_ = 2.67, *p* = 0.008; Figure 5C) and FCN-based (Discovery: *t*_134_ = 33.20, *p* < 0.001; Validation: *t*_134_ = 5.64, *p* < 0.001; Figure 5D) models were able to discriminate LDH-PCs from HCs. However, neither model was able to predict pain intensity in LDH-PCs (Validation: *p* = 0.812 and *p* = 0.605). In addition, the FCN-based model had greater discrimination accuracy than the mFC-based (75.9% vs. 64.3% AUC, Figure 5E), but lesser than MSN (80.1% AUC Figure 3C). The top 0.1% of linkages are illustrated in Figure 5F and detailed in Supplementary Table S4, respectively, which covered linkages between superior and middle frontal, precuneus, inferior parietal, pars opercularis, para-, pre- and postcentral, and inferior temporal cortex.

### 3.6 Performance of the ensemble model combined both morphometric features and resting-state signals

**FIGURE 6:**
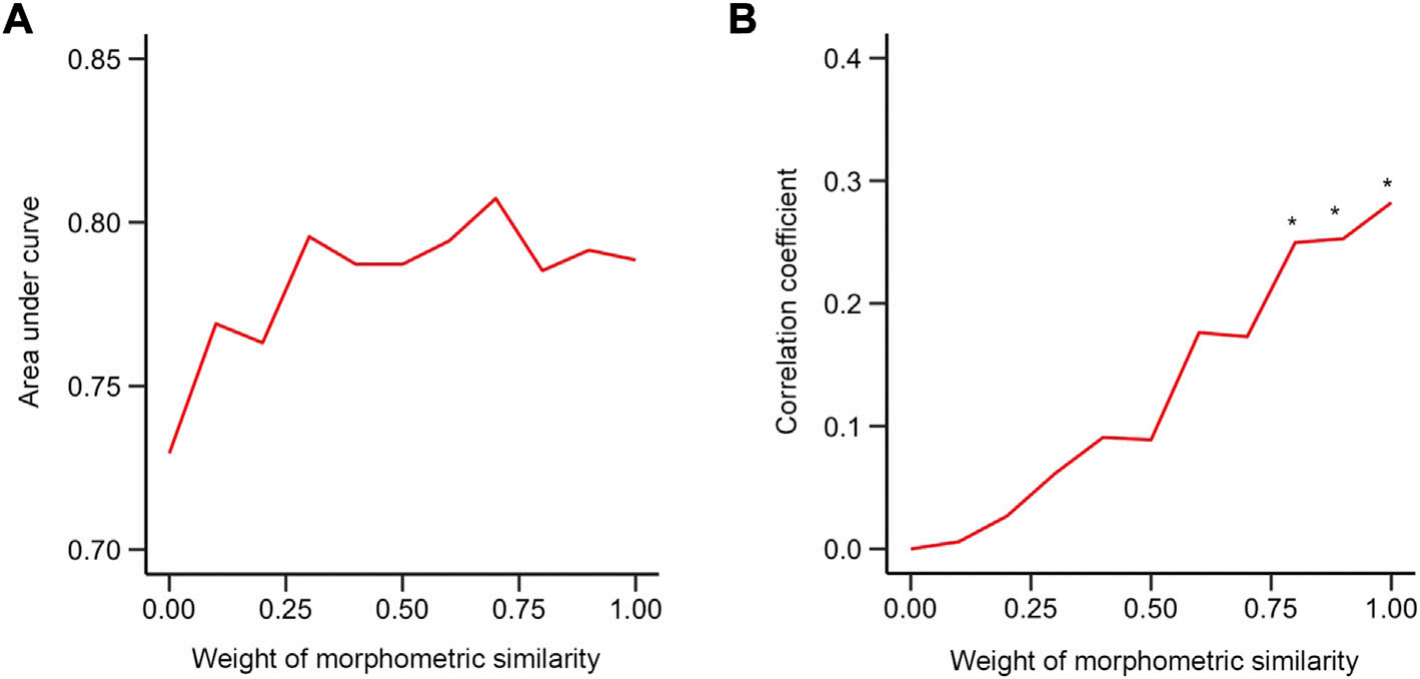
Performance of the mixed model **(A)** The relationship between AUC and weight of morphometric similarity in the validation group. **(B)** The relationship between the correlation coefficient between dot product and pain intensity of patients and weight of morphometric similarity in validation group. * indicates that the correlation at this weight was statistically significant compared to a null model of *r =* 0 (*p* < 0.05). (0: functional connectivity only; 1: morphometric similarity network only).

AUC was used as a criterion to evaluate the performance of the ensemble network model that combined both morphometric features and BOLD signals for discriminating LDH-CP patients from HCs and predicting pain intensity of LDH-CP patients. As shown in Figure 6A, as the weight of MS varied from 0 to 1, the range of AUC (Validation dataset) was between 0.7 and 0.8, indicating that for discriminating LDH-CPs from HCs, the discrimination ability of the ensemble network was high, and the changes in discrimination performance were minor when the weight of MS was changed. However, for predicting pain intensity, only greater MS weights (*w*_*ms*_ ≥ 0.8) had statistically significantly improved prediction performance (Figure 6B). Compared to FCN, MSN-based model not only discerns LDH-CPs from HC, but also predicts pain intensity of LDH-CPs.

## 4 Discussions

In this paper, we used morphometric similarity to study LDH patients with CP. We reveal that 1) MS-based models were able to discriminate LDH-CPs from HCs and the MSN model performed best; 2) MSN was able to predict the pain intensity of LDH-CPs; 3) functional connectivity networks constructed from the same template were able to discriminate LDH-CPs from HCs, but they could not predict pain intensity; and 4) an ensemble model, which combined both with morphometric features and resting-state signals, neither improved discrimination nor pain prediction performance.

To the best of our knowledge, this study is the first to successfully apply MS to CP data. This robust approach, which estimates the inter-subregional correlations using multimodal structural MRI variables in individual subjects (Seidlitz et al., 2018) has the capacity to extract different anatomical indices from both gray matter and tractography features. The finding from the mMS-based univariable model of the frontal polar [(−24, 56, 14); Supplementary Figures S1A,B] similarity abnormality as the only subregion that could discriminate the pain state of LDH-CPs from HC duplicates the previous findings of abnormalities in this subregion (Rodriguez-Raecke et al., 2009; Ong et al., 2019) in a new manner. Furthermore, MSN’s ability to discriminate between LDH-CPs and HCs and predict pain intensity demonstrates that MSN is sensitive enough to uncover brain morphology alterations associated with chronic pain (at least for LDH-CP patients). Unlike structural covariance analysis, which is based on a single morphometric feature (e.g., GM density) across subjects to construct anatomical “networks” (Baliki et al., 2011), MS quantifies the similarity in terms of multiple features within a subject, representing very different constructs. Thus, MS may pave a new way to explore anatomical brain alterations in disease states in general and specifically in chronic pain.

The top 0.1% of cortical regions that distinguished LDH-CP from HC are in frontal, precuneus, anterior cingulate, insula, pre- and postcentral, parahippocampal, and middle temporal cortex. Most of these regions overlap with those found by Baliki et al. (Baliki et al., 2011), where decreased GM density compared to HCs was found in frontal, insula, secondary somatosensory pre- and postcentral cortex, hippocampus, and temporal lobes. This regional overlap is unlikely coincidental, especially considering that the studies used two different neuroanatomical atlases (308-subregion template converted from Desikan-Killiay Atlas (Desikan et al., 2006) vs. 82-subregion template converted from WFU_Pick Atlas (Maldjian et al., 2003)), two different approaches (linkages from MSN vs. ROI-based GM density from univariable comparison), and were applied to two different CP populations (LDH-CP vs. CBP). Instead, these overlapping regions must be reflective of brain regions commonly changing for different types of chronic pain.

There is an ongoing debate whether pain is related to local brain subregions or is broadly “represented” within and across brain networks (Baliki and Apkarian 2015). Our data are more consistent with the latter. The three MS-based models for distinguishing LDH-PCs from HCs reveal that the more information integrated into the model, the better the discrimination (AUC: 80.1%, 64.1%, and 59.0% for MS network, 308 subregions, 1 subregion, respectively, Figure 3C). For LDH patients with chronic pain, the brain morphological differences associated with chronic pain not only arose from local subregions defined in the 308-subregion template, but from highly interconnected subregions. However, while LDH-CPs could be differentiated from HCs through the MSN model, and the intensity of associations between subregions and their locations (***β***_***upper***_, Figure 3B; Supplementary Table S2) could be identified, it remains unknown how these associations are specifically related to the neuropathology underlying chronic pain (Tracey and Bushnell 2009). More data from different chronic pain conditions need to be incorporated in future investigations to reveal the similarities and differences between types of chronic pain.

The success of distinguishing patients from HCs and predicting the pain intensity of LDH-CPs naturally inspired our curiosity of how a model based on functional connectivity network performed when using the same 308-subregion template that was transformed from Desikan–Killiany atlas. Although the Desikan–Killiany atlas is not based on functional ROIs or parcellations (Power et al., 2011; Gordon et al., 2016), we found that the FCN-based model had similar performance to that of the MSN-based one for distinguishing LDH-CPs from HCs (AUC: 80.1%, MSN, Figure 3C; 75.9%, FCN, Figure 5E). In addition, we observed that MSN and FCN only shared 5.6% of their respective variance (Figure 5B), indicating that MSN and FCN were relatively independent on the 308-subregion template. As observed in other clinical chronic pain conditions (Barroso et al., 2020; Barroso et al., 2021), chronic pain contributes to both neocortical morphological and functional connectivity reorganization. For LDH-CP patients, both MSN and FCN networks can be considered a pain-related state signature, reflecting patients’ pain history and accompanying negative emotion, anxiety, and depression (Baliki and Apkarian 2015). However, as we pushed further to investigate if an ensemble of the two networks would improve the performance of the discrimination, the results were not as favorable as we expected (Figure 5B). One reason might be that both models were based on an anatomical template, and moreover, the best model may not be a simple weighted average of functional and anatomical parameters. Questions remain regarding if and how a combination of MSN on the 308-subregion and FCN on a functional template would improve the discrimination, which requires future studies.

Studies have reported that morphological differences in patients with chronic pain were associated with pain duration as well as intensity (Geha et al., 2008; Wang et al., 2022). A meta-analysis of 39 studies demonstrated that pain duration correlated with gray matter in the insula, striatum, putamen, and amygdala, and right anterior cingulate gyrus (positive relationship) and frontal gyrus (negative relationship), respectively (Wang et al., 2022). Considering the observed relationship between the dot product derived from MSN and pain intensity (Figure 4A), MSN seems sensitive to morphological alterations (Seidlitz et al., 2018). Despite our large sample sizes, we failed to find statistically significant associations between dot products derived from the MSN model and pain duration, in addition to the interaction between pain intensity and pain duration (Geha et al., 2008). The failure may be attributed to different consequences of neuronal plasticity due to pain intensity and pain duration. Pain intensity can be understood as the magnitude of experienced pain and subtle neuronal changes associated with the experience could be caught by the morphometric similarity mapping; i.e., inter-subregion pair-wise correlation within an individual subject. However, pain duration may indirectly lead to neuronal plasticity associated with pain-related suffering and negative moods; the neuronal change is estimated by contrasting to matched healthy controls or performing linear regression within a group while the neuronal plasticity changes are more affected by age rather than pain duration. In other words, pain duration may be confounded and dominated by age effects.

An important limitation in this study is that we did not further explore two beta maps that relate to pain state (LDH-CPs vs. HCs) and pain intensity. For example, the correlation between local beta clusters and pain-related components [pain emotion, pain intensity, and pain sensitivity (Huang et al., 2019)]. The local beta values may be linked to these components. In the future, we plan to take advantage of our chronic pain database (https://openpain.org) to investigate local differences in beta maps across different chronic pain conditions by applying MSN models. Another limitation is relatively small sample sizes in this study that can increase variability of prediction (Varoquaux, 2018; Yang et al., 2020) and the conclusions drawn from the RS-FC analyses have not been corroborated in other pain clinical conditions while we have these image data, which will be done in the future.

## 5 Conclusion

With a large sample of LDH-CP patients and an unbiased validation strategy, MS mapping demonstrated to be a robust approach to distinguish brain morphological differences in patients with chronic pain. Three MS-based models were able to discriminate LDH-CPs from HCs and predict pain intensity of LDH-CPs with a PCA-LASSO-GLM model. The MSN-based model performed best among the three models. In addition, the relationship between MSN and functional connectivity derived from RS-fMRI was investigated on the same neuroanatomical template. FCN had similar performance to MSN for discriminating patients from controls but failed to predict pain intensity. An ensemble model combining both MSN and FCN did not appreciably improve the performance. Generally, MSN provides additional information to increase our understanding of the neuropathology of CP patients.

## Data Availability

The dataset in this manuscript is part of a multiphase project which is still under investigation. The data will eventually be made available in openpain.org once the project is complete. Reasonable requests can be sent to the corresponding authors.

## Supplementary material

Supplementary Figure S1 Mean MS in left rostral-middle-frontal-part8 can discriminate pain state of LDH-CP patients. **(A)** *t-*statistics of regional LDH-HC differences in the Discovery group. **(B)** left rostral-middle-frontal-part8 was the only subregion with a mean MS that statistically significantly discriminated (uncorrected *p* < 0.05) LDH-PC patients from HC; the result was replicated in validation group [centroid coordinates in MNI152 space: (−24, 56, 14)]. **(C)** Violin plots displayed the mean MS (red point) and its standard error bar of the left rostral-middle-frontal-part8 in discovery (left, *t*_134_ = 2.25, *p* = 0.025) and validation (right, *t*_134_ = 2.01, *p* = 0.047) groups.

Supplementary Figure S2 ROC curves of three models for discovery data after the Discovery and Validation groups were switched.

Supplementary Table S1 Information of the top 10% of betas from the mean MS across 308 subregions. Colored row indicates the subregion morphometric similarity of which was increased (red) or decreased (blue) compared with HCs (*p* < 0.05).

Supplementary Table S2 Information of the top 0.1% betas from the MSN model.

Supplementary Table S3 Information of the top 0.1% of betas that predicts pain intensity.

Supplementary Table S4 Information of the top 0.1% of betas from the FCN model.
