# Supplementary figures and images for: Morphometric similarity networks discriminate patients with lumbar disc herniation from healthy controls and predict pain intensity
Source: Front Netw Physiol. 2022 Oct 25;2:992662. doi: 10.3389/fnetp.2022.992662 (PMC10013053; doi:10.3389/fnetp.2022.992662)

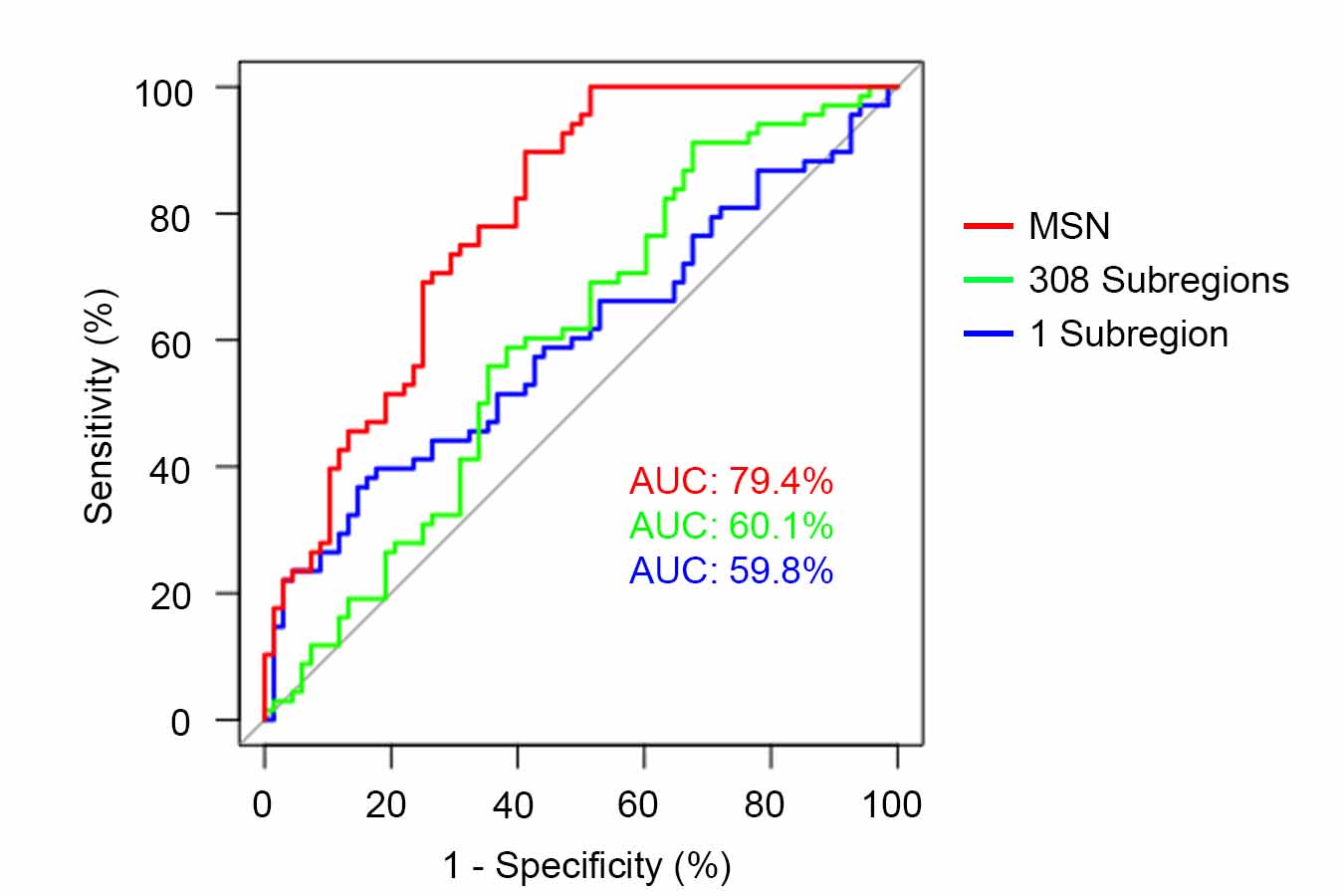

Supplement: Supplementary file 3 [file Image2.jpg]

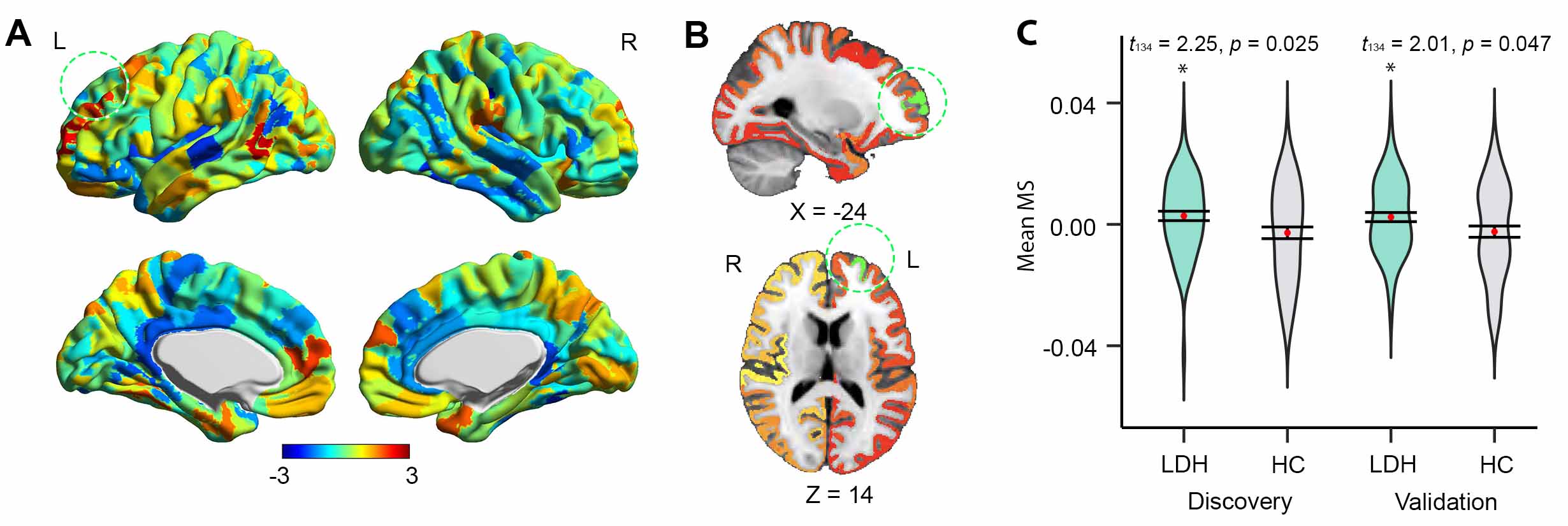

Supplement: Supplementary file 6 [file Image1.jpg]
